# Objectively measured physical activity and sedentary behaviour and ankle brachial index: Cross-sectional and longitudinal associations in older men

**DOI:** 10.1016/j.atherosclerosis.2016.01.038

**Published:** 2016-04

**Authors:** Tessa J. Parsons, Claudio Sartini, Elizabeth A. Ellins, Julian P.J. Halcox, Kirsten E. Smith, Sarah Ash, Lucy T. Lennon, S. Goya Wannamethee, I-Min Lee, Peter H. Whincup, Barbara J. Jefferis

**Affiliations:** a UCL Department of Primary Care & Population Health, UCL Medical School, Rowland Hill Street, London, NW3 2PF, UK; b UCL Physical Activity Research Group, UK; c Institute of Life Sciences, Swansea University, Singleton Park, Swansea, SA2 8PP, UK; d Brigham and Women's Hospital, Harvard Medical School, Boston, MA02215, USA; e Population Health Research Institute, St George's University of London, Cranmer Terrace, London, SW17 0RE, UK

**Keywords:** Physical activity, Ankle brachial index, Peripheral arterial disease, Epidemiology, Accelerometer, Men

## Abstract

**Background:**

Associations between bouts of physical activity (PA), sedentary behaviour (SB) and cardiovascular disease, and their mutual independence are not well defined. A low ankle brachial index (ABI ≤0.9) indicates peripheral arterial disease (PAD) and is predictive of cardiovascular events and functional impairment. We investigated the independence of PA and SB and the importance of bout duration in relation to ABI using objective measures.

**Methods:**

945 men from the British Regional Heart Study, mean age 78.4 y, had concurrent measurements of ABI (Vicorder) and physical activity (Actigraph GT3X accelerometer); 427 men also had accelerometer measurements one year previously and contributed data to longitudinal analyses.

**Results and conclusion:**

In cross-sectional analyses, after adjusting for covariates each extra 10 min of moderate and vigorous PA per day was associated with an OR of 0.81 (95% CI 0.72, 0.91) for a low ABI, a stronger association than for light PA (OR 0.85, 95% CI 0.75, 0.98). Each extra 30 min of SB was associated with an OR of 1.19 (95% CI 1.07, 1.33) for a low ABI. Associations between moderate and vigorous PA and ABI persisted after adjustment for light PA or SB. Bout lengths for PA and SB were not associated with a low ABI. One year changes in PA or SB were not associated with low ABI.

All physical activity and lower levels of SB, regardless of bout duration were inversely associated with ABI; more intense PA showed a stronger association. No associations between changes in PA and ABI were observed, but power may have been limited.

## Highlights

• Physical activity (PA) and ABI were objectively measured in British men (mean age 78y).
• PA was associated with a lower odds of a low ABI (OR 0.81, 95% CI 0.72, 0.91).
• Sedentary behaviour (SB) was associated with a higher odds (1.19) of a low ABI.
• Higher intensity PA showed a stronger association with ABI than lighter intensity PA.
• Total time spent in PA or SB, but not duration of bouts was associated with ABI.

## 1 Introduction

Whilst there is good evidence for higher moderate to vigorous physical activity (MVPA) levels and lower levels of sedentary behaviour (SB) reducing the risk of cardiovascular disease [1], little is known about the importance of activity bout length, how often sedentary behaviour should be interrupted, and whether light activity has health benefits in older age groups. There are few studies with objective measures of physical activity which allow more detailed investigation of these patterns. Peripheral arterial disease (PAD) is under-recognised compared with other cardiovascular diseases, and yet is the most common cause of major amputation [2] and is associated with functional impairment [3] and functional decline even among asymptomatic individuals [4]. Patients with PAD have high rates of fatal and non-fatal cardiovascular events, comparable to rates for patients suffering acute stroke or myocardial infarction [2], [5], and up to a third experience pain on walking (intermittent claudication) [6] and about half report atypical leg symptoms that interfere with mobility [7].

The ankle brachial index (ABI) is the ratio of ankle:arm systolic pressure and a non-invasive vascular measure that is predictive of cardiovascular events, independent of existing risk factors [8]. ABI is generally 1.0–1.4 in healthy individuals; values lower than 1, and particularly under 0.9, indicate progressively worse levels of peripheral arterial disease (PAD) [9]. A low ABI (≤0.9) has been associated with an approximately two-fold increase in 10 year total mortality, cardiovascular mortality and major coronary event rates, across all categories of baseline risk [8]. Two large recent studies, one cross-sectional [10], one longitudinal [11], both in middle aged adults (mean age 63y and 61y respectively) demonstrated associations between higher or increased self-reported physical activity levels and lower risk of PAD (as indicated by ABI<0.9). However some studies with self-reported physical activity have found associations only in women [12] or men [13] or no association [14]. Self reports of physical activity are limited in some respects, and tend to be less reliable in older adults [15]. Objective measures of physical activity allow more detailed investigation of patterns of physical activity and sedentary behaviour (SB, also a risk factor for CVD) so that different intensities of activity (light, moderate, vigorous) and SB can be quantified not only in terms of total amounts, but also time spent in bouts of different durations. Very few studies have examined objectively measured physical activity (PA) and SB in relation to ABI [16], [17], [18], one in a general population [16], and to our knowledge, questions around bout length and PA intensity have not yet been addressed.

Using a large sample of community-dwelling older men, we investigated associations between objectively measured physical activity of different intensities, moderate and vigorous activity (MVPA), light activity (LPA) and sedentary behaviour (SB), and PAD, as indicated by a low ABI (≤0.9). We also investigated whether the duration of bouts of activity (as indicated by current physical activity guidelines) was important, and whether the associations of PA or SB with ABI were independent. In addition, we examined longitudinal relationships between changes in MVPA, LPA and SB over 1 year and ABI.

## 2 Research design and methods

### 2.1 Sample

The British Regional Heart Study is a prospective, population-based cohort study following up 7735 men (>99% Caucasian) recruited from primary care practices in 24 British towns in 1978–80. In 2010–2012, 3137 survivors were invited to a physical examination which included measurement of ankle brachial index (ABI). In addition, all men were asked to wear a physical activity monitor (accelerometer) at yearly intervals since 2010, one of which coincided with the 2010–2012 physical examination. The National Research Ethics Service (NRES) Committee London provided ethical approval. Participants provided informed written consent to the investigation in accordance with the Declaration of Helsinki.

### 2.2 Ankle brachial index (ABI)

Ankle brachial index (ABI) was assessed on the right and left sides using a Vicorder (Skidmore Medical Ltd, Bristol, UK) with the participants supine. Hokanson SC10 cuffs were positioned on the upper arm and lower leg (above the ankle). Photoplethysmography sensors were clipped to the end of the middle finger and the big toe. The cuffs were inflated to 180 mmHg simultaneously occluding the brachial and tibial arteries. Blood pressures were taken at the point of the pulse returning at both sites as the cuffs slowly deflated. Two measurements were normally made for each side and the mean taken, but if the difference in sequential brachial and ankle recordings was >5 mmHg, three measures were taken for each side and a mean taken. Measurements from men whose vessels did not occlude were excluded. ABI was categorised as; low ≤0.9, borderline >0.9 and <1, normal 1.0–1.4, high >1.4 [9]. Men with either or both left and right values ≤0.9 were classified low and men with either or both values >1.4 were classified as having a high ABI. Men with ABI >1.4 were excluded from analyses since this usually indicates arterial calcification in the leg, which artificially increases ABI. All measures were made by 2 vascular technicians, with an intra-class correlation of 0.65.

### 2.3 Body mass index and blood pressure

Body mass index (BMI, kg/m^2^) was calculated from height (Harpenden stadiometer) and weight in light indoor clothing (Tanita body composition analyser (BC-418) or Tanita scales if the participant had a pacemaker or defibrillator). The average of two seated blood pressure readings (Omron HEM-907 recorder, mmHg) were used.

### 2.4 Objective physical activity assessment

Men wore the GT3X accelerometer (Actigraph, Pensacola, Florida) over the right hip for 7 days, during waking hours, removing it for swimming or bathing. Data were processed using standard methods [19]. Non-wear time was excluded using the R package “Physical Activity” [20]. Valid wear days were defined by convention as ≥600 min wear time, and participants with ≥3 valid days were included in analyses. Each minute of activity was categorised using intensity threshold values of counts per minute developed for older adults: <100 for sedentary behaviour (SB) (<1.5 MET), 100–1040 for light activity (LPA) (1.5–3 MET) and >1040 for moderate and vigorous physical activity (MVPA), (≥3 MET) [21].

### 2.5 Questionnaire data

Men self-completed a questionnaire including information about: current cigarette smoking, alcohol consumption, living alone, current use of antihypertensive medication, ever receiving a doctor diagnosis of heart attack, heart failure or stroke (with symptoms lasting >24 h), narrowing or hardening of the leg arteries (including claudication) (PAD) and leg pain on walking. Diabetes was defined as a doctor diagnosis, or a fasting blood glucose of ≥7 mmol/l. Social class was based on longest held occupation at study entry (1978–80) and categorised as manual and non-manual [22]. Region of residence (1978–80) was grouped into Scotland, North, Midlands and South of England.

### 2.6 Statistical methods

Men reporting a diagnosis of heart attack, heart failure, or stroke (with symptoms lasting >24 h) were excluded from analyses. Descriptive statistics for demographic characteristics, vascular measures, PA and SB, were calculated by category of ABI.

Associations between each of the different PA measures and ABI were investigated in a series of logistic regression models. The PA exposures investigated were: total activity counts per day, steps per day and minutes per day of SB, LPA and MVPA. For ease of interpretation the OR for a low ABI was estimated for each 10,000 counts of total activity, 1000 steps, 30 min of SB or LPA and 10 min of MVPA. In order to evaluate the independence of associations of activity intensities, models were mutually adjusted; (i) MVPA and SB and (ii) MVPA and LPA in the same model. SB and LPA were not included in the same model due to collinearity (r = −0.62). Associations between number of minutes accumulated in bouts of MVPA, LPA or SB of particular lengths were investigated with the following bout durations: SB lasting 1–15, 16–30, 31–60, and ≥61 min, LPA lasting 1–9 and ≥10 min, and MVPA lasting 1–9 and ≥10 min. Durations of MVPA were chosen to reflect current guidelines [23] but in the absence of recommendations for SB and LPA, durations were chosen based on their distributions. All models were adjusted for the following measurement-related factors (average accelerometer wear time (minutes/day), season of accelerometer wear (warm, May–September or cold, October–April), age, region of residence) and confounders (social class, living alone, smoking status and alcohol consumption). Further adjustments were made for BMI, systolic blood pressure, use of anti-hypertensive medication and use of statins.

Finally, a sub-group of men had physical activity data at two time-points; time 2 coincided with the physical examination (all measures) and time 1 (PA only) was approximately 1 year earlier. We investigated the relationship between change in PA/SB (time 1 to time 2) and odds of a low ABI at time 2. Logistic regression models included mean activity (mean of time 1 and time 2) and change in activity (time 1 to time 2) and were adjusted for both mean and change in accelerometer wear time, mean age, number of days between time 1 and time 2, season (3 categories; cold at both time-points, warm at both time-points, different at each time-point), region of residence, social class, living alone, smoking and alcohol consumption.

### 2.7 Sensitivity analyses

MVPA minutes were right skewed, so regression models were repeated using square root transformed MVPA to normalise the distribution. Models were also repeated after (i) combining men with a low (≤0.9) and men with a borderline (0.9–1.0) ABI so that men with values <1.0 were compared with men with values 1.0–1.4, (ii) excluding men with diabetes, (iii) excluding men reporting diagnosed PAD, and (iv) excluding men with diagnosed PAD and leg pain on walking.

## 3 Results

3137 men were invited to the physical examination; 1722 (55%) attended, of whom 1572 had an ABI measure. Measurement was not undertaken for some men due to health issues, e.g. swollen ankles, inability to tolerate the cuffs. Among men with a measure, 203 were excluded because their vessels did not occlude, and 42 excluded due to an ABI>1.4. Of the remaining 1327 men, 1130 had data for physical activity and all covariates. Of these, 185 men with pre-existing heart attack, heart failure, or stroke were excluded. 945 men contributed data to cross-sectional analysis and 427 men to longitudinal analysis. Of men who were invited to the examination, those with complete data and included in our study had a lower BMI 10 years earlier, 26.5 vs. 27.2 kg/m^2^, and were more active, 60% vs. 48% at least moderately active, than those who did not attend or did not have complete data.

Of men with cross-sectional data, 76 (8%) had a low ABI (Table 1). 80% of men had 7 days of accelerometer data and 96% had ≥5 days of data. Mean daily wear time was 857 min, accelerometer counts were 168,284 and steps were 5053. Men spent on average 613, 203 and 41 min per day in SB, LPA and MVPA respectively (72%, 24% and 5% of their day). Men with a low ABI (≤0.9) were more likely than men with a normal/borderline ABI (>0.9–1.4) to be of manual social class, smoke, take anti-hypertensive medications, have diabetes, self-report a doctor diagnosis of PAD, and self-report pain or discomfort in the leg(s) when walking (p < 0.05). Age, living alone, alcohol consumption or BMI did not relate to ABI (Table 1).

In regression models adjusted for only age and wear time (Table 2 Model 1), and in fully adjusted models (Table 2 Model 2), PA was associated with a lower, and SB with a higher odds of a low ABI. Each extra 10 min of MVPA per day was associated with an OR of 0.81 (95% CI 0.72, 0.91) for a low ABI, and each extra 30 min of LPA with an OR of 0.85 (95% CI 0.75, 0.98) (Table 2, Model 2). Each extra 30 min of SB was associated with an OR of 1.19 (95% CI 1.07, 1.33) for a low ABI (Table 2, Model 2). When MVPA and SB, or MVPA and LPA were mutually adjusted, the associations between MVPA and ABI persisted, whereas those between SB or LPA and ABI did not (Table 2, Models 1 and 2). SB and LPA were highly correlated (r = −0.62), and therefore not included in the same model. Including additional adjustments for BMI and systolic blood pressure (Table 2, Model 3) or use of anti-hypertensive medication or statins (data not presented) did not change our results.

### 3.1 Sensitivity analyses

Associations were essentially unchanged when models were repeated using square root transformed MVPA, when low and borderline ABI were combined (ABI <1), or when men with diabetes or PAD were excluded (data not presented). Excluding men with diabetes, PAD and leg pain on walking resulted in slightly stronger associations between LPA and low ABI and between SB and ABI, and whilst the association between MVPA and low ABI persisted with borderline significance when mutually adjusted for LPA, it did not when adjusted for SB (Supplementary Table 1).

### 3.2 Bouts of SB and PA

Few men accumulated many bouts of MVPA of ≥10 min; 31% accumulated 5 bouts per week, 5% accumulated 15 bouts per week. Total activity level (mean CPM) was correlated with bouts of MVPA lasting 1–9 or ≥10 min (r > 0.75) and bouts of LPA lasting 1–9 min (r = 0.63), weakly correlated with longer LPA bouts of ≥10 min (r = 0.33) and short SB bouts of 1–15 min (r = 0.21), and inversely correlated with longer SB bouts of 31–60 min (r = −0.40) and ≥61 min (r = −0.45) (p < 0.0001 for all coefficients). Minutes accumulated in bouts of MVPA lasting 1–9 min were associated with a lower odds of a low ABI (OR 0.98, 95% CI 0.96, 0.99) (Table 3, Model 1). The association for bouts of MVPA lasting ≥10 min was similar, although the confidence interval was slightly wider (OR 0.99, 95% CI 0.96, 1.01) (Table 3, Model 1). A Wald test indicated that the ORs did not differ from each other (p = 0.6). The associations between bouts of MVPA and ABI were little changed when bouts of LPA or SB were included in the model (Table 3, Models 4, 5), although odds ratios were no longer significant when MVPA and SB were included (Table 3, Model 4). For SB, time accumulated in bouts of 1–15 min, its inverse correlate, bouts of ≥61 min, and also bouts of 16–30 min were all associated with a higher odds of a low ABI (Table 3, Model 3), but confidence intervals were widened on adjustment for bouts of MVPA (Table 3, Model 5). Bouts of LPA were not significantly associated with ABI (Table 3, Model 2).

### 3.3 Longitudinal analyses

In the subset of 427 men with longitudinal data, the mean time between physical activity measures was 327 days (10.8 months). 22 (5%) men had a low ABI, and 25 (6%) men had a borderline ABI (>0.9 and <1). The changes in time spent in MVPA, LPA and SB between time 1 and time 2 were small. The mean change in percentage of time per day spent in MVPA was −0.3% (SD 2.5%), LPA −0.1% (4.2%) and SB 0.41% (5.3%) (percentages account for changes in wear time). Whilst mean counts, steps, MVPA and SB (mean of time 1 and time 2) were associated with a low ABI, change in PA variables or SB were not (Table 4); the ORs for change in counts, steps and MVPA were similar to the ORs for mean of levels at time 1 and time 2, but the confidence intervals for change were wider, so that they did not reach significance, likely the consequence of limited statistical power.

## 4 Discussion

To our knowledge, this is the first study to investigate, using objective PA measures, (i) the importance of shorter versus longer bouts of physical activity or sedentary behaviour and (ii) the relative importance of different intensities of PA, in relation to a low ABI (indicating PAD), and therefore makes a novel contribution to the existing literature. The prevalence of PAD increases with age, and global increases in life expectancy are leading to increasing numbers of people living with PAD, particularly among those aged over 80 [24]. The prevalence of low ABI (<0.9) in our study was 9%, a little lower than the estimates of 14% for 75–79 year olds and 16% for 80–84 year olds in other high income countries [24] or the 14% of ≥70 year old men in the NHANES study (1999–2000) [25]. Physical activity is recognised as a treatment strategy for patients with PAD, improving walking ability and walking distance [26], and is beneficial to a number of the risk factors for PAD, including being overweight, increased blood levels of total and low-density lipoprotein cholesterol and triglycerides, hypertension, diabetes [27], and endothelial function [28]. However the possible role of PA in the prevention of PAD remains under-studied and unquantified.

We found that the association between higher levels of MVPA and ABI was independent of LPA or SB, whereas associations between LPA or SB and ABI were not independent of MVPA, suggesting that more vigorous activity might be more protective than lighter activity against PAD. In the largest study to date, stronger beneficial associations between more intense activities (albeit self-reported) and ABI than less intense (e.g. running compared with walking) across a range of different activities were also reported [10]. We are aware of only three previous studies relating objective measures of physical activity to PAD indicated by a low ABI, one population based [16], the others restricted to samples with PAD [17] or diabetes [18]. All three studies reported potentially beneficial associations between PA and ABI although methodological differences prevent direct comparison of effect sizes. One study included sedentary time and also found that SB and MVPA (defined with different cut-points) were not independently associated with ABI ≤0.9 [16]. LPA was not investigated in these studies. We did not investigate whether associations between LPA and ABI were independent of SB or vice versa because LPA and SB were strongly inversely correlated; as SB increased, LPA decreased so it was not possible to include both in one model. Current UK physical activity guidelines for older adults recommend at least 150 min of moderate activity accumulated in bouts of 10 min or more, and that sitting for extended periods should be minimised. Prior studies of ABI have not investigated the importance of duration of bouts of activity [16], [17], [18]. In our study we did not find that bouts of MVPA ≥10 min were more strongly associated with ABI than bouts of MVPA <10 min. Likewise associations between SB and ABI were not stronger for longer bouts.

Findings from our longitudinal analyses were similar to those from the cross-sectional analyses in that the mean total activity, steps, MVPA and SB at time 1 and time 2 were associated with a lower (for PA) or higher (for SB) odds of a low ABI, and the associations were of a similar magnitude to those of the cross-sectional analyses. However, we did not find evidence that the change in any of the PA/SB variables was associated with a low ABI; although estimates for the change coefficients (except for LPA) were in the expected direction, confidence intervals were wide. We are aware of only one other longitudinal study, in which intentional activity (consciously done for exercise) protected against ABI falling to ≤0.9 over 3 years, from a baseline level of 0.9–1.4 [11]. In comparison, our follow-up period was relatively short (mean 11 months) and men in our study a decade older, which in addition to a reduced sample size for longitudinal analyses and modest changes in PA, likely explains why we did not see associations between change in PA/SB and ABI. The mechanisms through which PA may influence ABI have been reviewed elsewhere [29] and include improvements in nitric oxide–dependent vasodilation and mitochondrial energetics, possible increases in collateral flow, and decreases in systemic inflammation.

### 4.1 Strengths and limitations

Our study, for the first time, tested the importance of specific bout lengths of PA which relate to current physical activity guidelines. Our study sample was community dwelling men rather than a clinical group which increases generalisability and is a less-studied older age group. However findings may not be generalizable to younger ages or women. Although men who participated in our study were healthier than those who did not, we still have a wide range of activities represented in our sample (indicated by large standard deviations for activity variables in Table 1), so that any bias in average level of activity shouldn't affect our estimation of the associations between activity and ABI. We used an accelerometer which is validated for measuring low levels of energy expenditure but did not have good inclinometer data to determine whether men were standing or sitting during periods of <100 CPM. However, the mean value of these minutes was <10 CPM, suggesting that SB time was very sedentary and unlikely to include much standing time (particularly in the age range 70–90 years). Furthermore, varying the definition of SB from <100 to <50 CPM changed the total SB time very little, so any biases are likely to be small. Actigraph-measured SB has been demonstrated to have minimal bias compared to thigh-worn Activpal monitor measured SB (which differentiates sitting and standing with an inclinometer), and the two measures of SB correlate r = 0.76 [30]. We did not separate moderate and vigorous activity because a validated accelerometer cut point for older adults is lacking, the amount of moderate and vigorous activity in this age group is very small, and presenting these activities combined allows comparison with other studies. Our response rate for agreeing to wear an accelerometer (51%) [19] was greater than reported in other UK studies in older adults [31], [32], [33]. Adherence to the 7 day accelerometer wear protocol in our study was very good, with 96% of men providing the ≥5 days of data needed to predict habitual PA/SB [34].

We were able to adjust for a range of potential confounding factors, including important risk factors for PAD, such as smoking, diabetes and blood pressure, although we are unable to establish the direction of effect from our cross-sectional analyses. We present longitudinal analyses although they were limited with respect to sample size and length of follow up. It is known that patients with PAD have lower physical activity levels [17], which may well be a consequence of subclinical atherosclerosis rather than a cause, but the associations between higher PA levels and lower odds of a low ABI remained after excluding men with leg pain on walking, suggesting that a reduction in physical activity due to leg pain did not bias our findings.

## 5 Conclusions

Our study suggests that total amounts of MVPA are associated with a lower risk of PAD, indicated by ABI ≤0.9. Higher levels of LPA and lower levels of SB were also associated with a lower risk of PAD, but not independently of MVPA. However, we did not find that longer bouts of MVPA, LPA or SB were more strongly associated with ABI than shorter bouts. Few older people meet the physical activity guidelines; 15% of our sample accumulated the recommended 150 min of MVPA in bouts of at least 10 min, although 62% of men accumulated 150 min of MVPA in bouts of 1 min or more [19]. Encouraging all moderate and vigorous activity regardless of bout length might make goals more attainable for this low active age group and help them to increase activity and lower their risk of PAD.

## Funding

This work was supported by the British Heart Foundation [PG/13/86/30546 and RG/13/16/30528] and the National Institute of Health Research [Post-Doctoral Fellowship 2010–03–023]. IML was partly supported by National Institutes of Health [CA154647]. The funders had no role in the design and conduct of the study; collection, management, analysis, interpretation of the data; preparation, review, approval of or decision to publish the manuscript. The views expressed in this publication are those of the author(s) and not necessarily those of the Funders.

## Conflict of interest

None declared.

## Figures and Tables

**Table 1:** Characteristics of 945 men without pre-existing CVD or heart failure, by ABI.

Mean (SD) or % (n)	Low ABI <0.9	Normal/borderline ABI ≥0.9–1.4	P	All men	N
N	76	869			945
Age (years)	78.8 (4.2)	78.2 (4.5)	0.23	78.2 (4.5)	945
Manual Social class,% (n)	61.8 (47)	43.5 (378)	0.002^a^	45.0 (425)	945
Lives alone, % (n)	21.1 (16)	18.2 (158)	0.54^a^	18.4 (174)	945
Smoker, % (n)	15.8 (12)	2.9 (25)	<0.0001^a^	3.9 (37)	945
Taking anti-hypertensives, % (n)	67 (51)	52 (454)	0.013^a^	53.4 (505)	945
Alcohol (units/week)	6.0 (6.7)	6.6 (7.8)	0.46	6.6 (7.8)	945
Body mass index (kg/m^2^)	27.1 (3.9)	26.9 (3.6)	0.66	26.9 (3.6)	939
Systolic blood pressure (mmHg)	146.4 (16.9)	147.6 (20.7)	0.56	146.5 (17.2)	943
Diabetes, % (n)	22 (17)	11 (98)	0.005^a^	12.2 (115)	945
Peripheral arterial disease, % (n)	14.5 (11)	2.0 (15)	<0.0001^a^	2.8 (26)	945
Leg pain, % (n)	45 (34)	25 (221)	<0.0001​a	27.0 (255)	945
Wear time (mins/day)	844 (72)	858 (67)	0.08	857 (67)	945
Total activity (counts per minute)	118,473 (64,238)	172,640 (96,073)	<0.0001	168,284 (95,037)	945
Steps (mean/day)	3554 (1829)	5185 (2713)	<0.0001	5053 (2689)	945
% time spent sedentary	75.9 (8.1)	71.2 (9.2)	<0.0001	71.6 (9.2)	945
SB (min/day)	640 (84)	610 (85)	0.003	613 (85)	945
LPA (min/day)	178 (57)	205 (64)	<0.0001	203 (64)	945
MVPA (min/day)	25 (21)	43 (33)	<0.0001	41.4 (32.1)	945

ABI, ankle brachial index.

SB, sedentary behaviour.

LPA, light physical activity.

MVPA, moderate and vigorous physical activity.

a Pearson chi square test.

**Table 2:** Cross sectional associations between physical activity, sedentary behaviour and low ABI, men aged 70–91y, n = 945.

Daily physical activity	Model 1		Model 2		Model 3	
OR	(95% CI)	OR	(95% CI)	OR	(95% CI)
Vertical counts (/10,000/day)	**0.92**	**(0.88,0.95)**	**0.93**	**(0.89,0.97)**	**0.92**	**(0.88,0.96)**
Steps (/1,000/day)	**0.73**	**(0.64,0.83)**	**0.77**	**(0.67,0.87)**	**0.74**	**(0.65,0.86)**
MVPA (/10 min/day)	**0.78**	**(0.69,0.88)**	**0.81**	**(0.72,0.91)**	**0.79**	**(0.70,0.90)**
LPA (/30 min/day)	**0.82**	**(0.72,0.94)**	**0.85**	**(0.75,0.98)**	**0.85**	**(0.74,0.97)**
SB (/30 min/day)	**1.23**	**(1.11,1.37)**	**1.19**	**(1.07,1.33)**	**1.21**	**(1.08,1.35)**
Mutually adjusted analyses						
MVPA (/10 min/day)	**0.82**	**(0.70,0.95)**	**0.84**	**(0.71,0.98)**	**0.83**	**(0.70,0.97)**
SB (/30 min/day)	1.07	(0.93,1.24)	1.05	(0.91,1.22)	1.06	(0.91,1.23)
MVPA (/10 min/day)	**0.80**	**(0.70,0.91)**	**0.82**	**(0.72,0.94)**	**0.81**	**(0.71,0.93)**
LPA (/30 min/day)	0.93	(0.81,1.08)	0.95	(0.82,1.10)	0.94	(0.81,1.09)

Model 1: adjusted for average daily accelerometer wear time and age.

Model 2: adjusted for average daily accelerometer wear time, season of wear, region of residence, age, social class, living alone, tobacco, alcohol consumption.

Model 3 (n = 937): adjusted for average daily accelerometer wear time, season of wear, region of residence, age, social class, living alone, tobacco, alcohol consumption, systolic blood pressure, BMI.

Bold type indicates results significant at the 5% level.

ABI, ankle brachial index.

MVPA, moderate and vigorous physical activity.

LPA, light physical activity.

SB, sedentary behaviour.

**Table 3:** Cross sectional associations between bouts of physical activity, sedentary time, and ABI in 945 men aged 70–91y.

	Low ABI	N = 945
OR^a^	(95% CI)
**Model 1**		
MVPA mins in bouts 1–9 min	**0.975**	**(0.959,0.992)**
MVPA min in bouts 10+ min	0.986	(0.961,1.011)
Wald test^b^		0.60
**Model 2**		
LPA mins in bouts 1–9 min	0.994	(0.987,1.001)
LPA mins in bouts 10+ mins	0.997	(0.978,1.016)
Wald test^b^		0.80
**Model 3**		
SB mins in bouts 1–15 min	**1.011**	**(1.002,1.020)**
SB mins in bouts 16–30 min	**1.012**	**(1.004,1.021)**
SB mins in bouts 31–60 min	1.004	(0.998,1.011)
SB mins in bouts 61+ mins	**1.008**	**(1.003,1.013)**
Wald test b		0.18
**Model 4**		
MVPA mins in bouts 1–9 min	**0.977**	**(0.957,0.997)**
MVPA mins in bouts 10+ min	0.985	(0.960,1.011)
LPA mins in bouts 1–9 min	1.001	(0.992,1.009)
LPA mins in bouts 10+ min	0.993	(0.974,1.012)
**Model 5**		
MVPA mins in bouts 1–9 min	0.981	(0.958,1.005)
MVPA mins in bouts 10+ min	0.988	(0.962,1.014)
SB mins in bouts 1–15 min	1.003	(0.992,1.015)
SB mins in bouts 16–30 min	1.008	(0.999,1.018)
SB mins in bouts 31–60 min	0.999	(0.991,1.008)
SB mins in bouts 61+ min	1.003	(0.996,1.010)

Bold type indicates results significant at the 5% level.

ABI, ankle brachial index.

SB, sedentary behaviour.

LPA, light physical activity.

MVPA, moderate and vigorous physical activity.

a Adjusted for average daily accelerometer wear time, season of wear, region of residence, age, social class, living alone, tobacco and alcohol consumption.

b Wald test for odds ratios equal to each other.

**Table 4:** Associations between change in physical activity and sedentary time between time 1 and time 2, and ABI at time 2, in 427 men aged 71–90y at time 1.

	Low ABI	N = 427
OR^a^	(95% CI)
**Model 1**		
Change in counts (/10,000/day)	0.91	(0.82,1.02)
mean counts (/10,000/day)	**0.91**	**(0.84,0.99)**
**Model 2**		
Change in steps (/1000/day)	0.76	(0.52,1.10)
Mean steps (/1000/day)	**0.71**	**(0.53,0.94)**
**Model 3**		
Change in MVPA (/10 min/day)	0.79	(0.58,1.06)
Mean MVPA (/10 min/day)	**0.77**	**(0.60,0.99)**
**Model 4**		
Change in LPA (/30 min/day)	1.11	(0.74,1.67)
Mean LPA (/30 min/day)	0.79	(0.60,1.03)
**Model 5**		
Change in SB (/30 min/day)	1.02	(0.72,1.44)
Mean SB (/30 min/day)	**1.26**	**(1.02,1.56)**
**Model 6**		
Change in MVPA (/10 min/day)	0.74	(0.52,1.06)
Mean MVPA (/10 min/day)	0.84	(0.60,1.17)
Change in SB (/30 min/day)	0.86	(0.57,1.28)
Mean SB (/30 min/day)	1.11	(0.82,1.52)
**Model 7**		
Change in MVPA (/10 min/day)	0.78	(0.57,1.06)
Mean MVPA (/10 min/day)	0.81	(0.62,1.06)
Change in LPA (/30 min/day)	1.17	(0.78,1.76)
Mean LPA (/30 min/day)	0.90	(0.66,1.22)

​Bold type indicates results significant at the 5% level.

ABI, ankle brachial index.

SB, sedentary behaviour.

LPA, light physical activity.

MVPA, moderate and vigorous physical activity.

a Adjusted for average daily accelerometer wear time (mean of time 1 and time 2), change in daily accelerometer wear time (between time 1 and time 2), number of days between time 1 and time 2, season of wear, region of residence, age, social class, living alone, smoking and alcohol consumption.
