# Supplementary material for: Objectively measured physical activity and sedentary behaviour and ankle brachial index: Cross-sectional and longitudinal associations in older men
Source: Atherosclerosis. 2016 Apr;247:28–34. doi: 10.1016/j.atherosclerosis.2016.01.038 (PMC4819952; doi:10.1016/j.atherosclerosis.2016.01.038)
Supplement: Supplementary file 1 [file mmc1.docx]

**Supplementary Table 1. Cross sectional associations between physical activity, sedentary behaviour and low ABI in men without diabetes, peripheral arterial disease and leg pain on walking, aged 70-91y, n=590**

|  | **Model 2** | |
| --- | --- | --- |
| **Daily physical activity** | **low ABI N=590** | |
|  | **OR^1^** | **(95% CI)** |
| Vertical counts (/10,000/day) | **0.93** | **(0.88,0.99)** |
|  |  |  |
| Steps (/1,000/day) | **0.76** | **(0.62,0.92)** |
|  |  |  |
| MVPA (/10 mins/day) | **0.81** | **(0.68,0.97)** |
|  |  |  |
| LPA (/30 mins/day) | **0.79** | **(0.63,0.98)** |
|  |  |  |
| SB (/30 mins/day) | **1.25** | **(1.05,1.49)** |
| Mutually adjusted analyses |  |  |
| MVPA (/10 mins/day) | 0.89 | (0.71,1.13) |
| SB (/30 mins/day) | 1.15 | (0.90,1.47) |
|  |  |  |
| MVPA (/10 mins /day) | **0.85** | **(0.71,1.03)** |
| LPA (/30 mins/day) | 0.87 | (0.68,1.11) |

^1^adjusted for average daily accelerometer wear time, season of wear, region of residence, age, social class, living alone, tobacco, alcohol consumption.

ABI, ankle brachial index

MVPA, moderate and vigorous physical activity

LPA, light physical activity

SB, sedentary behaviour
